# Integrated charge excitation triboelectric nanogenerator for all weather wave energy harvesting

**DOI:** 10.1016/j.isci.2026.115693

**Published:** 2026-04-13

**Authors:** Wenxuan Chang, Hengyu Guo

**Affiliations:** 1 State Key Laboratory of Mechanical Transmission, College of Mechanical and Vehicle Engineering, Chongqing University, Chongqing 400044, China; 2 School of Physics, Chongqing University, Chongqing 400044, China

**Keywords:** energy sustainability, energy application, devices

## Abstract

Triboelectric nanogenerators represent a highly promising technology for large-scale blue energy harvesting. TENGs have many advantages that make it promising for energy harvesting applications. However, the development of TENGs was hampered by structural design limitations. This work presents an oblate spheroidal triboelectric nanogenerator for all-weather blue energy harvesting. The device integrates three spring-steel-based units and employs a half-wave rectifier charge excitation circuit, where one-unit primes three main units to enhance overall output. We systematically investigate the working mechanism and output performance of the TENG at various frequencies. Moreover, the symmetrical oblate spheroidal shell structure ensures stable TENG output even under inversion conditions by leveraging its geometric stability. The high output power of this TENG configuration demonstrates significant potential for future all-weather blue energy harvesting applications.

## Highlights

• The spring steel sheet structure enables device stacking, increasing output
• With the introduction of a charge excitation mechanism, enhance the charge density
• The flat spherical shell structure ensures all-weather energy harvesting

## Graphical abstract

**Figure:**
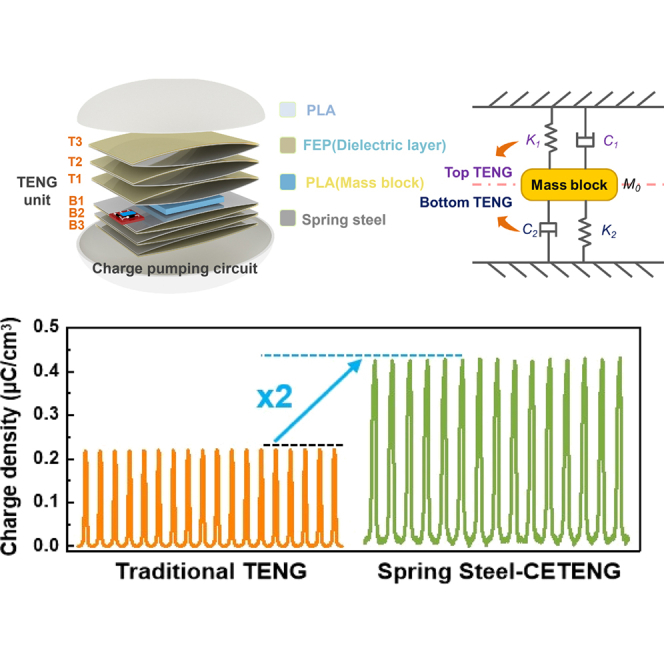

## Introduction

In recent years, triboelectric nanogenerators (TENGs) have garnered considerable interest and have been extensively applied in harnessing blue energy,^1^^,^^2^ wind energy,^3^^,^^4^ vibrations,^5^^,^^6^ and other forms of mechanical energy.^7^^,^^8^ As a new energy technology for self-power systems invented in 2012, TENGs have been demonstrated to have great application prospects in many fields,^9^ such as energy harvesting,^10^^,^^11^^,^^12^ active sensor devices,^13^^,^^14^^,^^15^ human-computer interaction,^16^^,^^17^ portable power source,^18^^,^^19^ and so on.^20^^,^^21^^,^^22^^,^^23^^,^^24^ TENGs provide benefits including lightweight design, affordability, simple construction, a diverse range of applicable materials, and high efficiency in capturing low-frequency mechanical energy.^25^ Different from conventional generators, TENG uses triboelectrification and electrostatic induction to convert mechanical energy into electrical energy.^26^

In general, TENG is categorized into four primary working modes: contact-separation mode,^27^ single electrode mode,^28^ in-plane sliding mode,^29^ and free-standing mode.^30^ Based on the four basic modes of operation described above, a variety of different structures of TENG have been prepared for specific application scenarios.^31^^,^^32^ Through material optimization, device structure design, and process optimization, the performance is greatly improved, and the output power is increased from several watts to several hundred watts per square meter.^33^^,^^34^ Through the power management circuit, the energy storage efficiency is greatly improved, and the maximum energy utilization efficiency can reach 85%.^35^ The output power and energy of the TENG are directly linked to the density of the triboelectric surface charge. Therefore, increasing the output charge density is essential for driving more powerful electronic devices continuously with miniaturized TENG, which can save cost and facilitate integration.^36^^,^^37^ This pursuit of higher charge density and output performance is particularly critical for harvesting large-scale, low-frequency, and distributed energy sources, such as ocean wave (blue) energy.

Blue energy, which is extensively distributed worldwide, is considered an essential renewable resource.^38^ The TENG was created to capture ambient mechanical energy, paving the way for a new area of energy conversion and applications.^39^ It holds significant promise for development as a renewable energy source, given that ambient energy, particularly mechanical energy, is widespread and nearly limitless, as shown by various energy harvesting devices.^40^^,^^41^ The TENG operates on the principles of the triboelectric effect and electrostatic induction, demonstrating high output performance.^32^^,^^42^^,^^43^ TENG is particularly effective for harvesting low-frequency energy,^44^^,^^45^ making it highly suitable for collecting blue energy.^46^^,^^47^ Herein, we present an oblate spheroidal TENG, elaborately designed with two contact - separation modes. It comprises multiple basic units based on spring steel plates, optimized for all-weather blue energy harvesting. In such a working principle, we use a half-wave rectifier to obtain the maximum energy output. In this study, a 30 μm thick fluorinated ethylene propylene film, uniformly overlaid with a spring steel sheet, was utilized as both springs and electrodes in a foldable TENG, effectively capturing wave energy from the ocean and converting it into electricity. The working mechanism and key factors affecting the output performance of TENG are analyzed systematically. The open-circuit voltage (V_OC_) of the CE-TENG is 5.5 kV with a half-wave rectifier circuit, resulting in a transferred charge (Q_SC_) of 1.82 C. These findings highlight the strong potential of the CE-TENG for harnessing blue energy.

## Results

### Structure and working principle of CE-TENG

**Figure 1:**
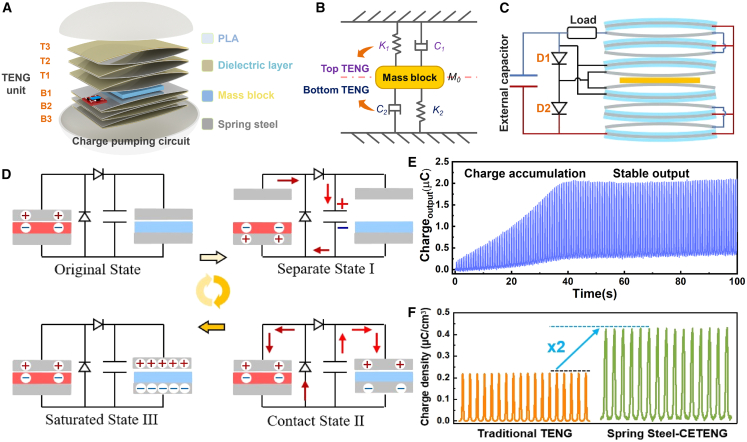
Structure schematic and working mechanism of the CE-TENG (A) 3D structural diagram of the CE-TENG. (B) The equivalent vibration schematic of the model can be considered as a two-spring-damping system. (C) A schematic diagram of the structure of the device. (D) The working mechanism of CE-TENG. (E) The output charge accumulation by the half-wave rectifier circuit. (F) Variation of charge under different materials TENG.

Figure 1A shows the 3D structure of the device, which consists of 2 parts: the TENG unit and the oblate spheroidal shell. The CE-TENG has two symmetrical parts, T1 and B1, which are the excitation TENG, and the other is the main TENG. Due to their excellent elastic deformation ability, fast response to pressure, and excellent physicochemical properties, the spring steel sheets provide stable durability and are well-suited as supports and metal electrodes for TENGs. The device consists of a spring steel sheet (the length of 80 mm, the width of 50 mm, the thickness of 0.10 mm) as the support and electrode, and a 30 μm thick FEP as the friction layer, forming a contact separation mode TENG. A half-wave rectifier circuit is used for charge accumulation. The CE-TENG is achieved through excitation TENG transfers charges to the capacitor, where they are accumulated and stored. Subsequently, the accumulated charges are cyclically transferred between the main TENG and the capacitor, thereby enhancing overall charge accumulation. Through multi-layer structure design, higher accumulation of charges can be achieved faster. Figure 1B is a conceptual diagram of the structure, where the contact-separation motion of the TENG is achieved by collecting vibration energy to compress and stretch the spring steel sheet. This motion is analogous to the vibration of a spring. Figure 1C is a schematic diagram of the structure of the device. Through the symmetrical structure to achieve the collection of vibration energy, the up and down vibration of the middle counterweight block, compression and stretch symmetrical TENG, a symmetrical structure to ensure the stable operation of the device in a harsh environment, such as flip tilt. The Working Principle of TENG is shown in Figure 1D; the output energy of the TENG is increased by a half-wave rectifier circuit. In the original state (Figure 1D and i), the excitation TENG is in contact. Subsequently, the excitation TENG generates a charge to external capacitors through the half-wave rectifier circuit (Figure 1D and ii). After that, when the TENG is in contact again, the charge stored in the capacitor is transferred to the main TENG to reach an equilibrium state. The main TENG generates an alternating current due to electrostatic induction and contact charging (Figure 1D and iii). The charge stored in the main TENG flows back to the capacitor due to the change in capacitance of the main TENG. The system reaches stability after several working cycles. Under the cyclic motion, the charge is continuously transferred from the excitation TENG to capacitors and reaches saturation, and the output reaches a maximum. The main TENG achieves a high charge density (Figure 1D and iv). Figure 1E shows the TENG output with the vibration energy collected by the half-wave rectifier circuit, and the output charge accumulation is stabilized. Compared to traditional TENG (Supporting Information Figure S1), the spring steel output with half-wave rectification achieves a 200% increase in body charge density, as shown in Figure 1F. It demonstrates that the spring steel TENG with half-wave rectification improves its output performance successfully.

### Output performance of the CE-TENG

**Figure 2:**
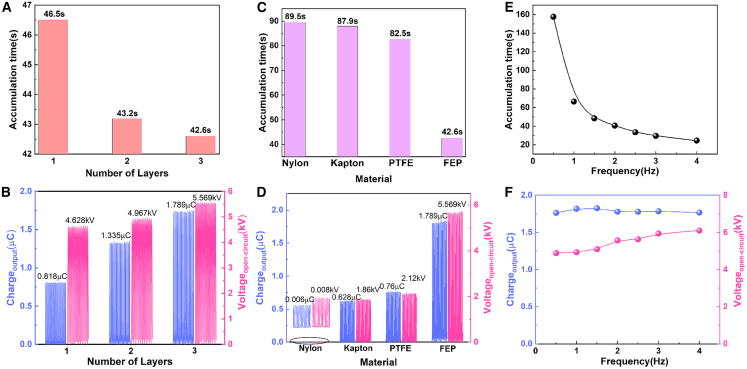
The performance of CE-TENG (A) Stable excitation time for CE-TENG across different layers. (B) The output of CE-TENG at different layers. (C) Stable excitation time for CE-TENG in different materials. (D) The output of CE-TENG in different materials. (E) Stable excitation time for CE-TENG at different frequencies. (F) The output of CE-TENG at different frequencies.

To characterize the electrical output performance of the TENG, variable frequency amplitudes are used to simulate ocean wave energy. The V_OC_, short-circuit current (I_SC_), and Q_SC_ of the excited TENG are measured under different frequencies. As the frequency increases, the output gradually rises. Since the frequency of ocean waves is generally 0.25–4 Hz, we chose 2 Hz for the experimental demonstration. To compare the output of different stack layers of the TENG, the output charge is measured under half-wave rectification. The output performance of the excitation TENG is shown in the Supporting Information Figure S2. As the number of device layers increases, the half-wave rectification excitation time gradually decreases and the output gradually increases, as shown in Figures 2A and 2B and Supporting Information Figures S3–S5, under excitations of 2 Hz. The charge output increases and stabilizes to 0.818 μC, 1.335 μC, and 1.789 μC, and the voltage output increases and stabilizes to 4.628 kV, 4.967 kV, and 5.569 kV, respectively. Figures 2C and 2D and Supporting Information Figure S6 display the transfer charge with different materials. The output performance of the charge excitation TENG with four types of materials is studied at 2 Hz. Due to the difference in the electronegativity of the materials, the accumulation time varied through the excitation circuit. Nylon requires 89.5 s, Kapton requires 87.9 s, PTFE requires 82.5 s, but FEP only needs 42.6 s to achieve a stable charge. It explains that the transfer charge is largest with FEP film due to a higher triboelectronegativity. The charge output increases and stabilizes to 0.006 μC, 0.628 μC, 0.76 μC, and 1.789 μC, and the voltage output increases and stabilizes to 0.008 kV, 1.86 kV, 2.12 kV, and 5.569 kV, respectively. The output of the TENG unit are measured and shown in Figures 2E and 2F. Devices of two-layer structure with FEP triboelectric layer were tested, the excitation circuit reaches the stabilization time, and gradually decreases with the gradual increase of the vibration frequency, while the transfer charge is basically unchanged, and the voltage increases with the increase of the frequency.

**Figure 3:**
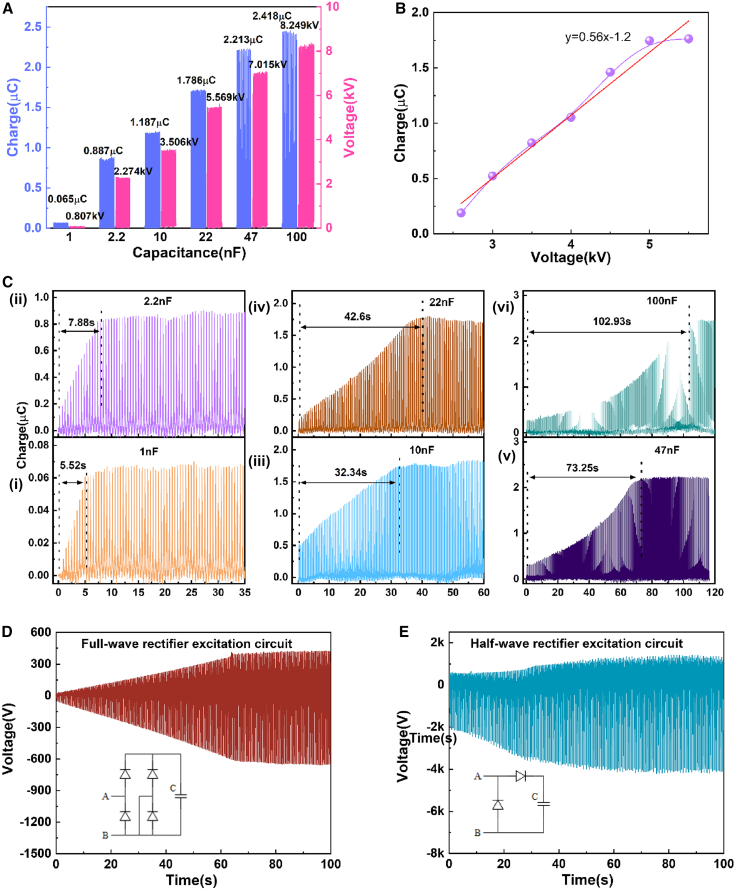
The performance of the excitation circuit (A) The output of different capacitors. (B) The relationship between transfer charge and voltage. (C) Charge accumulation curves for different capacitance values. (D) The voltage of the full-wave rectifier excitation circuit. (E) The voltage of the half-wave rectifier excitation circuit.

The effect of different capacitors in the excitation circuit on the CE-TENG output is systematically discussed later in discussion. Theoretically, the amount of charge transferred between the main-TENG and the capacitor gradually increases as the capacitor capacity increases, then approaches saturation. As shown in Figure 3A, the Q_SC_ and voltages when the excitation circuit reaches stability with different capacitors (1.0, 2.2, 10, 22, 47, and 100 nF, respectively). As the value of capacitance in the circuit increases, the output also increases. When a 47 nF capacitor is used for the excitation circuit, the relationship between transfer charge and voltage is shown in Figure 3B, where the transfer charge rises linearly with increasing voltage at the beginning and then saturates. The size of the capacitance in the excitation circuit determines the time it takes for the charge to reach saturation output; the larger the capacitance value, the longer the accumulation time and the more charge transferred. It is clear from the test that if the capacitance value is small, the Q_SC_ in each vibration cycle also decreases. Moreover, as the capacitance value increases, the saturation time for the excitation circuit to reach stabilization also increases. Under different capacitance values (1.0, 2.2, 10, 22, 47, and 100 nF, respectively) in the excitation circuit, complete charge accumulation curves are shown in Figure 3C, respectively. When using 1.0 nF capacitors, the saturated charge is 0.065 μC and the charge accumulation time is merely 5.52 s. As the capacitors gradually increase, the accumulation time also increases, and so does the saturation charge. At a 100 nF capacitor with a saturation charge of 2.418 μC, the charge accumulation time has increased to 102.93 s. The stabilization time gradually increased from 5.52 s to 102.93 s. Comparing the effect of full-wave rectification and half-wave rectification excitation circuits on the output voltage is shown in Figures 3D and 3E. In the excitation circuit, the time to reach stability is the same, and the output voltage of the full-wave rectification circuit reaches 1.1 kV, while the voltage output of the half-wave rectification circuit is up to 5.5 kV. The half-wave rectification has the advantages of a simple structure and high conversion efficiency. Under the same conditions, half-wave rectification always has a higher maximum energy than full-wave rectification.

**Figure 4:**
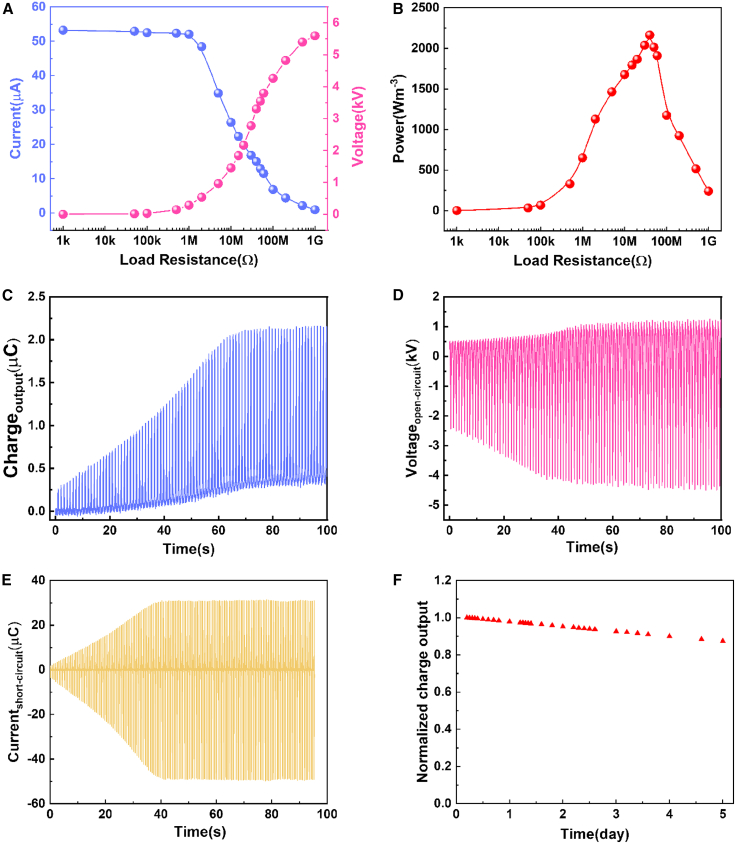
The performance of CE-TENG at 2 Hz (A) The current of the CE-TENG under various impedances. (B) The output power density under various impedances. (C) The charge at 2 Hz. (D) The voltage of charge at 2 Hz. (E) The current of charge at 2 Hz. (F) The stability of CE-TENG after 860,000 cycles at 2 Hz motion frequency for 5 days.

The output power density is shown in Figures 4A and 4B and, Supporting Information Video S1, where the load resistance varies from 1 KΩ to 1 GΩ at 2 Hz. As resistance increases, current decreases with the increase of load resistance, and voltage increases with the increase of load resistance. The maximum power density is 2163 W m^−3^ with a resistance of 40 MΩ at an operating frequency of 2 Hz; 480 green light-emitting diodes (LEDs) are lighted. The Q_SC_, I_SC_, and V_OC_ at 2 Hz operating frequency with 47 nF are displayed in Figures 4C–4E. The device yields a Q_SC_ of 2.213 μC, a I_SC_ of 56 μA, and an V_OC_ of 7.015 kV. The stability of TENG is important for its practical application to ensure that TENG can provide energy to electronic devices for a long time. The output performance of the CE-TENG retained 93% of its initial value after 860,000 cycles at 2 Hz motion frequency for 5 days, showing an excellent stability advantage (Figure 4F).

Video S1. The output power density under various impedances

### Application demonstrations of CE-TENG

**Figure 5:**
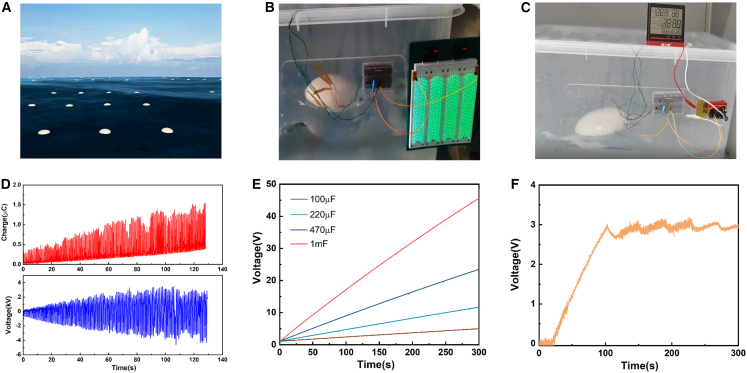
Application demonstrations of CE-TENG (A) Schematic of the multi-module CE-TENG network on the ocean surface. (B) Photograph of 480 green LEDs is lit by the CB-TENG at a 2 Hz wave vibration. (C) Photograph of a hygrothermograph powered by the CE-TENG at 2 Hz wave vibration. (D) The output voltage and charge of CE-TENG under water-wave vibration at 2 Hz. (E) The charging abilities of CE-TENG for different capacitors. (F) The corresponding voltage curve of the storage capacitor.

The multi-module TENG network realizes large-scale blue energy harvesting connected by a hexagonal network structure and floats the TENG in parallel on the ocean surface to supply energy to electronic devices, all-weather, as shown in the schematic diagram of Figure 5A. The CE-TENG converts ocean energy into electricity at a 2 Hz wave vibration. Charge accumulation gradually stabilizes by the half-wave rectifier circuit excitation. After the output is stable, 480 green LEDs are lighted as shown in Figure 5B and Supporting Information Video S2. A commercial temperature-humidity monitor is continuously driven by CE-TENG through a power management system (Supporting Information Figure S7) as shown in Figure 5C and Supporting Information Video S3. The output voltage and charge of CE-TENG under water-wave vibration at 2 Hz are shown in Figure 5D. The output charge and voltage grow rapidly, and then gradually stabilize. The capacitors of 100 μF, 220 μF, 470 μF, and 1 mF are charged to 4.8 V, 11.7 V, 23.6 V, and 45.6 V in 300 s, respectively, and are shown in Figure 5E. The real-time monitoring voltage-time curve is shown in Figure 5F and Supporting Information Video S4, the output voltage of CE-TENG reaches 3 V (operating frequency 2 Hz). When the temperature and humidity monitor starts to operate, the voltage reduces slowly, and then when the voltage drops to about 2.6 V, the TENG continues to work to stabilize the voltage. All of the above is strong evidence of the CE-TENG harvesting ocean energy and its great potential in the field of mechanical vibration.

Video S2. 480 green LEDs are lighted by the CB-TENG at 2 Hz wave vibration

Video S3. Hygrothermograph powered by the CE-TENG at 2 Hz wave vibration

Video S4. The real-time monitoring voltage-time

## Discussion

In this paper, contact-separated TENGs are designed through a spring steel sheet structure to effectively reduce the device volume to enhance the volume charge density. The CE-TENG is realized via a charge excitation circuit combined with integrated stacked TENGs, which synergistically enhance the output volume charge density. The electrical output performance of the TENG employing spring steel and a half-wave rectification circuit is twice that of a traditional TENG. The volume charge density is maintained at 170 μC cm^−3^. As the number of device layers increases, the output charge increases and stabilizes at 0.818 μC, 1.335 μC, and 1.789 μC, respectively. With a 47 nF capacitor at 2 Hz, the output reaches approximately 2.213 μC in Q_SC_, 56 μA in I_SC_, and 7.015 kV in V_OC_. Under the same conditions, Half-wave rectification always has a higher maximum energy than full-wave rectification. The maximum power density is 2163 W m^−3^ with a resistance of 40 MΩ at 2 Hz. The CE-TENG keeps more than 93% of its power output after more than 400 000 operating cycles. This work provides a new strategy for TENG to achieve ocean energy harvesting and high volume charge density in practical applications.

### Limitations of the study

The core design principle of CB-TENG is to harvest ocean wave energy. Performance characterization was conducted at 2 Hz, which is higher than the dominant frequency range of actual ocean waves (∼0.25–4 Hz). Therefore, the output power under real wave conditions may be lower than the result. Moreover, the experiments were performed in a simulated water tank, without accounting for long-term corrosion, biofouling, or multidirectional wave forces in real marine environments. Furthermore, although the charge excitation circuit enhances output, it may increase system complexity and energy management overhead. Finally, the work did not fully evaluate inter-unit coupling effects in large-scale arrays or long-term durability under harsh oceanic conditions. These aspects should be addressed in future research and practical deployment.

## Resource availability

### Lead contact

Further information and requests for resources and reagents should be directed to and will be fulfilled by the lead contact, Hengyu Guo (physghy@cqu.edu.cn).

### Materials availability

Materials used in the study are commercially available.

### Data and code availability

• All data reported in this paper will be shared by the lead contact upon reasonable request. Hengyu Guo (physghy@cqu.edu.cn).
• No new code was generated during the course of this study.
• Any additional information required to reanalyze the data reported in this paper is available from the lead contact upon reasonable request.

## Acknowledgments

This work is financially supported by the 10.13039/501100001809NSFC (52302219, 52572204), the Science and Technology Research Program of 10.13039/501100007957Chongqing Municipal Education Commission (KJZD-K202500505), 10.13039/501100005230and the Natural Science Foundation of Chongqing (CSTB2025NSCQ-GPX1032).

## Author contributions

W.X. Chang and H.Y. Guo conceived and designed the project. W.X. Chang and H.Y. Guo wrote the manuscript.

## Declaration of interests

The authors declare no conflicts of interest for this work.

## STAR★Methods

### Key resources table

**Table:** 

REAGENT or RESOURCE	SOURCE	IDENTIFIER
**Chemicals, peptides, and recombinant proteins**		

Spring Steel Plate	Kobetool, Germany, width 50 mm, thickness 0.10 mm	N/A
FEP film	Shanghai Witlan Industry Co., Ltd	N/A

### Experimental model and study participant details

This study does not use experimental methods typical in the life sciences.

### Method details

#### Fabrication of the CE-TENG components

The CE-TENG components were mainly composed of two parts. For the CE-TENG part, TENG consists of a spring steel plate as support and electrode and FEP as friction layer to form a contact separation mode friction nanogenerator. Commercial spring steel plate (Kobetool, Germany, width 50 mm, thickness 0.10 mm) was coiled and calcined in a 500 mL beaker with a radius of 90 mm at 400°C for 5 h, then cooled at room temperature to obtain a spring steel sheet with a radius of about 100 mm, cut to a length of 80 mm. Commercial FEP film (30 μm) as a friction layer, closely covering the spring steel sheet. For the package shell, a 3D printed ABS package shell sealed by AB adhesive (Shell radius 10 cm, thickness 5 cm).

### Quantification and statistical analysis

The unpackaged CE-TENG was driven by a linear motor (LinMot S01-72/500) at 0.5–4 Hz. A water storage tank was driven by linear motors to simulate the natural waves. The shortcircuit current and the transferred charge of the TENG were tested by an electrometer (Keithley 6514) with a Data Acquisition Card (NI PCI-6259), and the load voltage was measured by the partial pressure method.

### Additional resources

This study has not generated or contributed to a new website/forum.
