# Supplementary material for: Integrated charge excitation triboelectric nanogenerator for all weather wave energy harvesting
Source: iScience. 2026 Apr 13;29(5):115693. doi: 10.1016/j.isci.2026.115693 (PMC13138234; doi:10.1016/j.isci.2026.115693)
Supplement: Document S1. Figures S1–S7 [file mmc1.pdf]

iScience, Volume *29*

Supplemental information

Integrated charge excitation triboelectric nanogenerator for all weather wave energy harvesting

Wenxuan Chang and Hengyu Guo

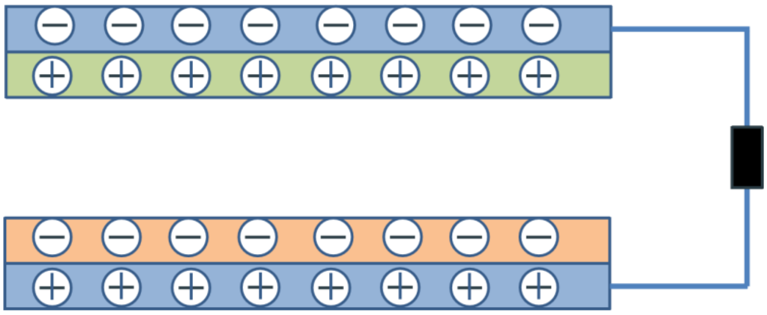

Figure S1 The "traditional TENG" structure

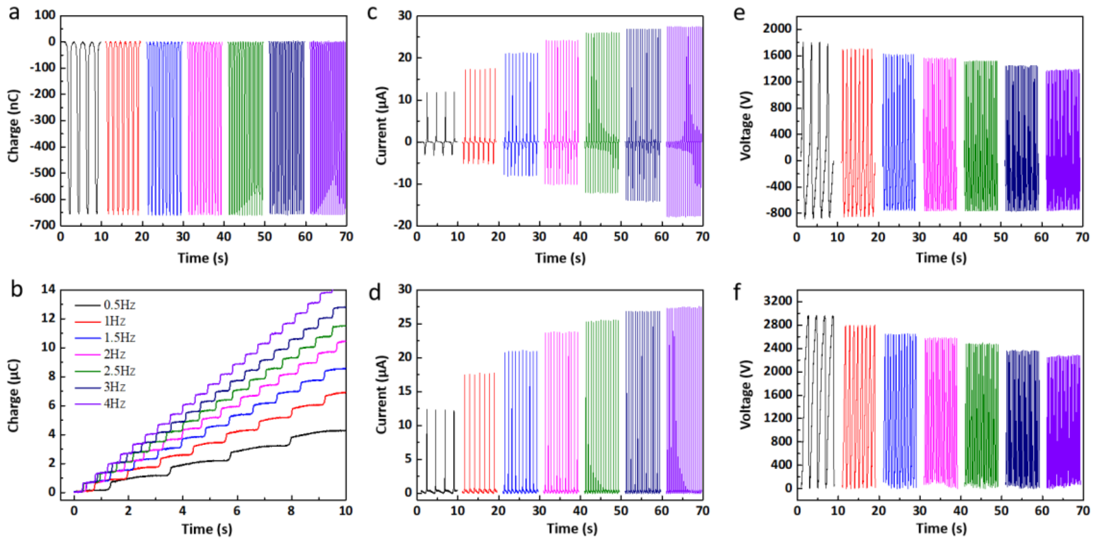

Figure S2 The output performance of the excitation TENG.

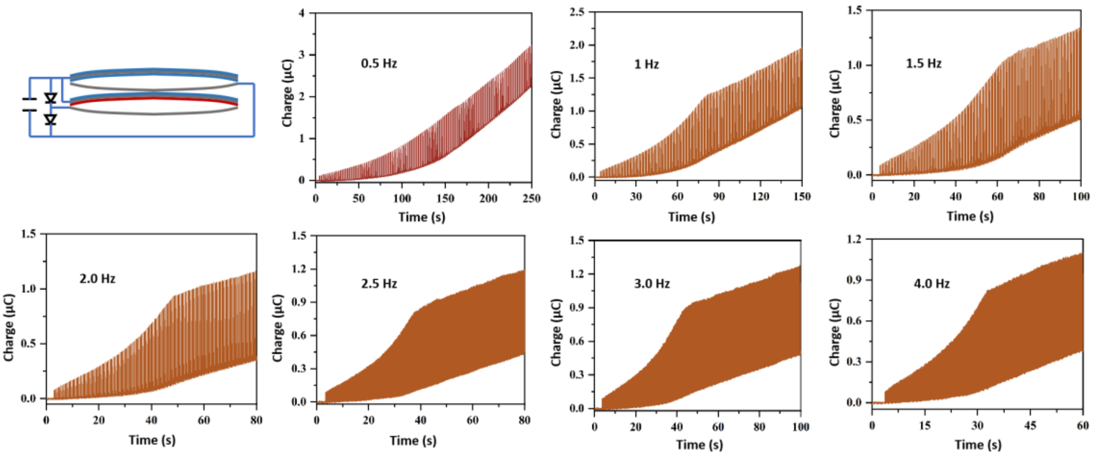

Figure S3 The charge output of single-layer devices at different frequencies.

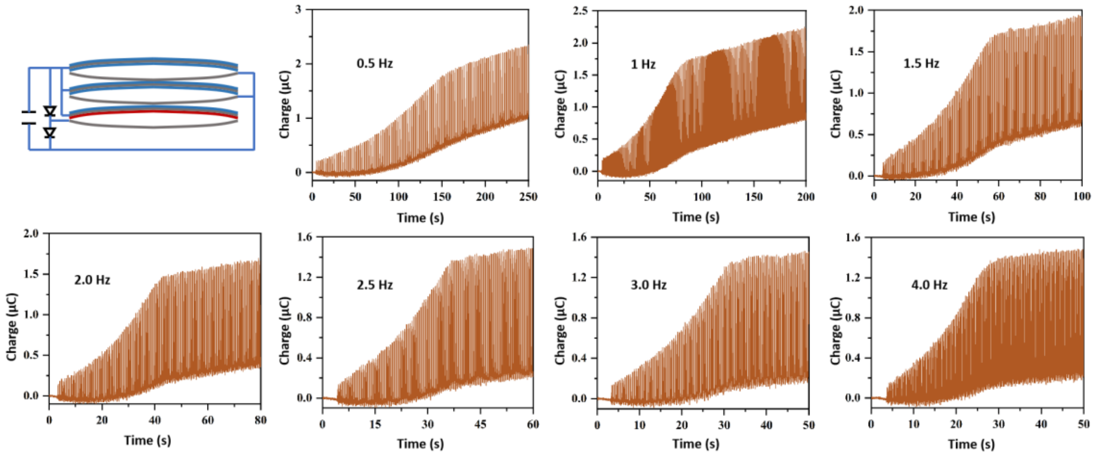

Figure S4 The charge output of the two-layer device at different frequencies.

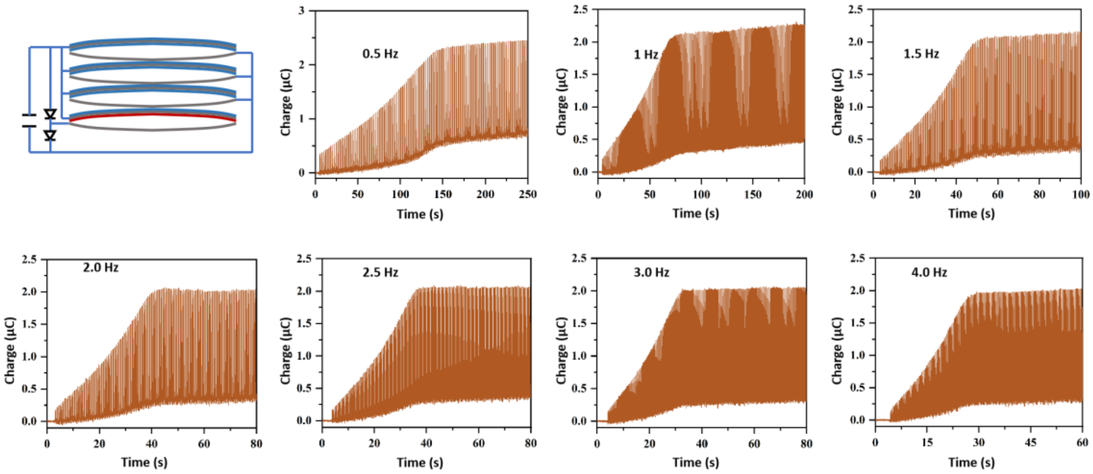

Figure S5 The charge output of the three-layer device at different frequencies.

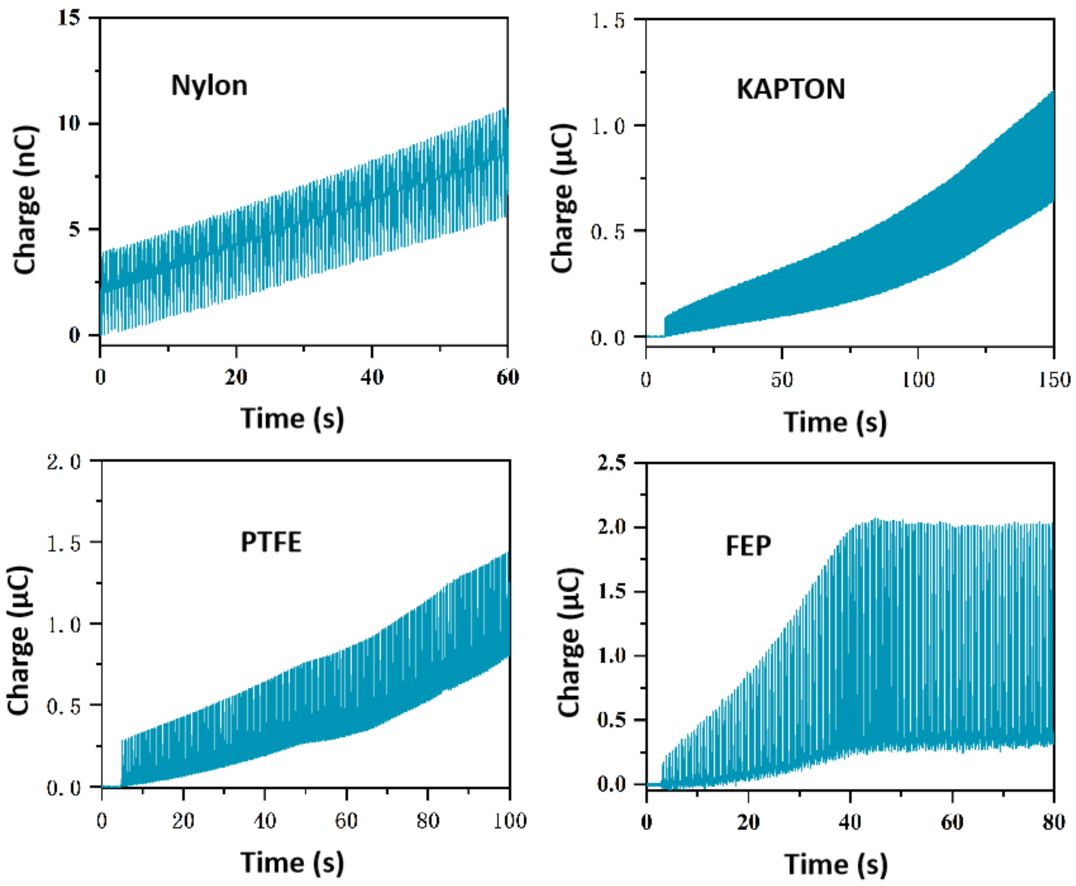

Figure S6 The charge output of different materials.

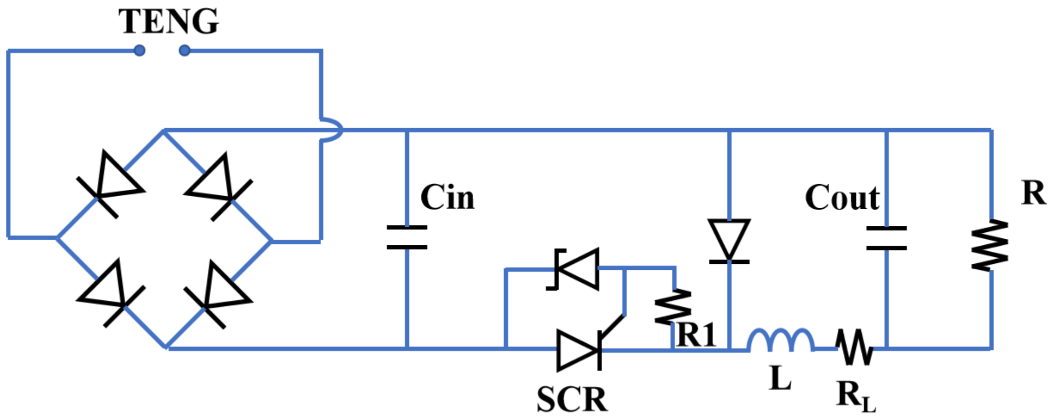

Figure S7 Thepower management system circuit diagram.
